# Clinical parameters and outcomes of necrotizing soft tissue infections secondary to gastrointestinal fistulas

**DOI:** 10.1186/s12879-019-4248-0

**Published:** 2019-07-09

**Authors:** Kun Guo, Wenbin Gong, Tao Zheng, Zhiwu Hong, Xiuwen Wu, Huajian Ren, Gefei Wang, Guosheng Gu, Peter Nthumba, Jianan Ren, Jieshou Li

**Affiliations:** 1 Department of General Surgery, Medical School of Nanjing University, Jinling Hospital, 305 East Zhongshan Road, Nanjing, 210002 Jiangsu Province People’s Republic of China; 2 Department of General Surgery, School of Medicine, Southeast University, Jinling Hospital, 305 East Zhongshan Road, Nanjing, 210002 Jiangsu Province People’s Republic of China; 3 AIC Kijabe Hospital, Kijabe, Kenya

**Keywords:** Necrotizing soft tissue infections, Gastrointestinal fistula, Outcome

## Abstract

**Background:**

Necrotizing soft tissue infections (NSTIs) is severe surgical infections which can occur following trauma or abdominal surgery. NSTIs secondary to gastrointestinal (GI) fistula is a rare but severe complication.

**Methods:**

A retrospective cohort study was performed on all subjects presenting with GI fistulas associated NSTIs were included. Clinical characteristics, microbiological profile, operations performed, and outcomes of patients were analyzed.

**Results:**

Between 2014 and 2017, 39 patients were finally enrolled. The mean age were 46.9 years and male were the dominant. For the etiology of fistula, 25 (64.1%) of the patients was due to trauma. Overall, in-hospital death occurred in 15 (38.5%) patients. Microbiologic findings were obtained from 31 patients and *Klebsiella pneumoniae* was the most common species (41.0%). Eight patients were treated with an open abdomen; negative pressure wound therapy was used in 33 patients and only 2 patients received hyperbaric oxygen therapy. Younger age and delayed abdominal wall reconstruction repair were more common in trauma than in non-trauma. Non-survivors had higher APACHE II score, less source control< 48 h and lower platelet count on admission than survivors. Multiple organ dysfunction syndrome, multidrug-resistant organisms and source control failure were the main cause of in-hospital mortality.

**Conclusions:**

Trauma is the main cause of GI fistulas associated NSTIs. Sepsis continues to be the most important factor related to mortality. Our data may assist providing enlightenment for quality improvement in these special populations.

## Background

Gastrointestinal (GI) fistula represents a relatively rare yet serious condition in clinical scene [1–3]. Despite recent advances in the field of surgical infections, the incidence and mortality of intestinal fistulas is particularly high [4–6]. Infectious complications secondary to GI fistula can range from localized infection to lethal sepsis. Even more serious is that once the uncontrolled spillage of intestinal content into the abdominal wound, with ongoing sepsis and usually accompanies extensive necrosis of the abdominal wall fascia [6]. There is no doubt that necrotizing soft-tissue infections (NSTIs) of the abdominal origin are a rare but serious in-hospital complication [7].

NSTIs are a group of life-threatening skin infections associated with high rates of both morbidity and mortality [8–10]. However, there are a few well-documented cases of NSTIs secondary to gastrointestinal fistula [11–13]. GI fistula associated NSTIs has special clinical characters compared to other types of NSTIs [14]. These critically ill surgical people’s conditions change rapidly, and there are serious disorders in the host homeostasis. Despite the severity of this complication, there is no generally accepted therapeutic approach to GI fistulas associated NSTIs. Brafa et al [15] reported that used an abdominoplastic advancement technique for delayed primary closure of a cancer patient affected by necrotizing fasciitis after 2 months of serial debridement and negative pressure wound therapy (NPWT).

However, most studies on GI fistulas associated NSTIs are retrospective case series and focus on predictors of mortality and length of hospitalization [16–18]. Very few studies have examined surgical procedures or clinical outcomes. We report a large series of NSTIs secondary to GI fistulas at a tertiary-referral center. This report analyzes the surgical treatment of GI fistula associated NSTIs, and also examines related factors that affect patient’s prognosis.

## Methods

Patients with GI fistula associated NSTIs at the Research Institute of General Surgery in Jinling Hospital between 2014 and 2017 were identified from the prospectively maintained gastrointestinal fistula database. The study was conducted in accordance with the ethical principles of the Helsinki Declaration and was approved by the Jinling Hospital Ethics Committee. Written informed consent was obtained from all individual participants included in the study. The patients included in the database have been followed by reviewing their medical information and by directly contacting the patients.

All patients were included in this study according to the following inclusion criteria: (1) age between 18 and 65 years; (2) diagnosis of GI fistula associated NSTIs according to clinical, radiological and histological findings. Patients who died or discharged within 48 h of admission was excluded. Patients with incomplete or missing date were also excluded.

### Patient characteristics

Data onto patient demographics (age, sex, BMI, primary disease), comorbidities (hypertension, diabetes mellitus, and obesity), and in-hospital management measures were extracted. Additional data were obtained from the computerized hospital medical records. For microbiologic examinations, wound drainage samples and cultures were obtained during each individual. We also recorded data on in-hospital management, length of stay (LOS) and in-hospital death.

### Definitions

The clinical diagnosis of NSTIs was made by attending surgical physicians, whereas clinical suspicion was validated via histological examinations [19, 20].

Sepsis and septic shock were diagnosed according to the standard criteria [21]. Sepsis is defined as evidence of infection plus life-threatening organ dysfunction, and a sequential organ failure assessment (SOFA) score ≥ 2. Septic shock is defined as sepsis plus persistent hypotension, requiring vasopressor to maintain mean arterial pressure (MAP) ≥65 mmHg, and serum lactate levels > 2 mmol/L despite adequate fluid resuscitation. Samples of microbial cultures were routinely collected when the patient had fever (> 38 °C) and there was evidence of clinical suspicion or infection.

Source control was performed according to the clinician’s assessment and established criteria [22]: resolution of fever, oral temperature < 37.5 °C; resolution of leukocytosis, white blood cells (WBC) < 12.0/10^9^ and absence of bands and immature neutrophil forms; resolution of physical findings of tenderness and rigidity and restoration of enteric function; no further operative or percutaneous intervention required.

### In-hospital management

**Fig. 1:**
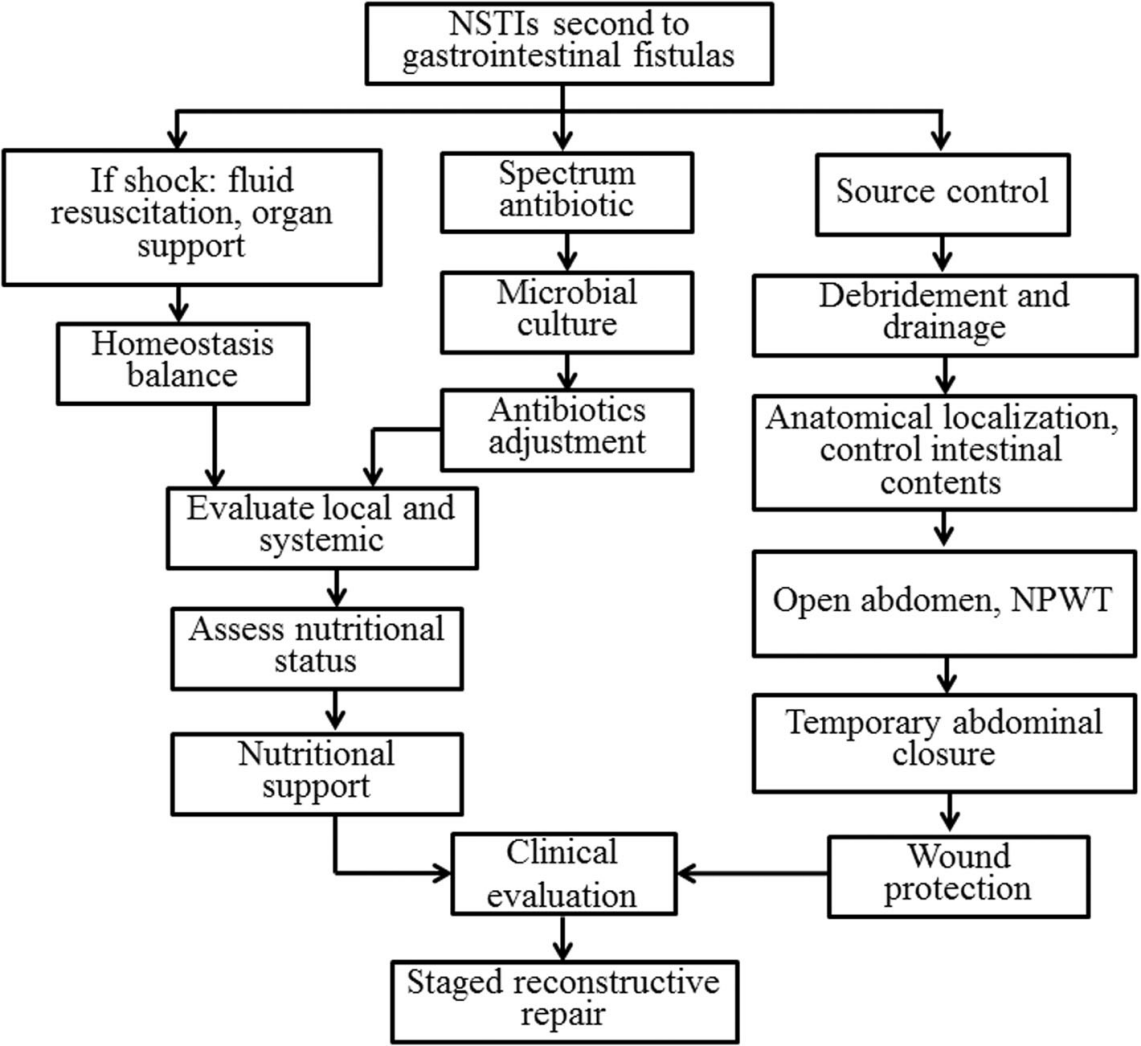
In-hospital management of GI fistula associated NSTIs. In our center, the management of GI fistula associated NSTIs were treated according to the following principles: severe sepsis and septic shock were managed by standard therapies; source control by percutaneous or surgical drainage (open abdomen if necessary); broad-spectrum antibiotics therapy was initiated in all patients; other supporting treatments as needed. After the patient’s systemic and local conditions have improved, staged reconstructive repair included digestive tract reconstruction and abdominal wall reconstruction would be performed

Because our hospital is a tertiary referral center of China, all fistula patients had undergone surgery at least once prior to admission into our center. In our center, the management of GI fistula associated NSTIs requires initial control of sepsis with drainage of source and the placement of drains to eliminate inflammation, as shown in Fig. 1. Briefly, all enrolled patients were treated according to the following principles: severe sepsis and septic shock were managed by standard therapies [23]; source control by percutaneous or surgical drainage (open abdomen if necessary); broad-spectrum antibiotics therapy was initiated in all patients; other supporting treatments as needed. After the patient’s systemic and local conditions have improved, stag reconstructive repair included digestive tract reconstruction and abdominal wall reconstruction would be performed.

### Statistical analysis

Statistical analyses were performed using SPSS 22.0 (SPSS Inc., Chicago, IL) for Windows. Categorical data are presented as numbers (proportion) and continuous data as means±standard deviation (SD). Differences of continuous data between groups were compared using Student *t* test or Mann–Whitney tests. Categorical variables were compared using χ ^2^ test or Fisher’s exact test. A two-sided *p* value < 0.05 was considered statistically significant.

Given the different extent of physiological derangement between trauma and non-trauma settings, we conducted subgroup analysis to compare the difference between trauma and non-trauma. To determine factors potentially associated with in-hospital death, we also conducted a subgroup analysis according to in-hospital outcome (survivors versus non-survivors).

## Results

### Baseline characteristics

Between 2014 and 2017, 39 patients satisfied the study inclusion criteria (Table 1). Thirty (76.9%) were male and 19 (23.1%) were female; 25 (64.1%) were post trauma and 14 (35.9%) non-trauma. The mean body mass index was 20.4 kg/m^2^ and the mean age were 46.9 years. Cardiac disease and renal insufficiency were found in one patient each. The main disease etiologies contributing to the fistulae were trauma (25, 64.1%), tumor (9, 23.1%) and other (5, 12.8%). Small intestinal fistula was the most common type (39.0%), followed by colorectal (30.5%) and duodenum fistula (11.9%). Multiple fistulas were found in 16(41.0%) patients. The mean C-reaction protein (CRP), WBC, platelet count and hemoglobin on admission was 121.6 (mg/L), 11.0(10^9^/L), 179.3 (10^9^/L) and 105.5 (g/dl). Overall, in-hospital death occurred in 15 (38.5%) patients.

**Table 1:** Demographic and clinical characteristics of patients with NSTIs secondary to gastrointestinal fistula (*n* = 39)

Variable	
Gender, (Male), n (%)	30 (76.9)
Age (years), mean (SD)	46.9 ± 16.9
BMI (kg/m ^2^), mean (SD)	20.4 ± 1.9
Co-morbidities, n (%)	7 (17.9)
Cardiac disefficiency	2 (5.1)
Renal disefficiency	2 (5.1)
Diabetes	1 (2.6)
Hypertension	1 (2.6)
COPD	1 (2.6)
Admission APACHE score, mean (SD)	11.4 ± 3.7
Shock on admission, n (%)	21 (53.8)
Positive blood cultures, n (%)	17 (43.6)
Cause of fistula, n (%)	
Trauma	25 (64.1)
Tumor	9 (23.1)
Other	5 (12.8)
Location of fistula (total number of fistula = 59), n (%)	
Small intestine	23 (39.0)
Colorectal	18 (30.5)
Duodenum	7 (11.9)
Stomach	4 (6.8)
Pancreas	3 (5.1)
Other	4 (6.8)
Multiple fistulas, n (%)	16 (41.0)
The flow of fistula (ml/24 h), n (%)	
< 200	1 (2.6)
200–500	11 (28.2)
> 500	27 (69.2)
The laboratory data on admission, mean (SD)	
C-reactive protein (CRP) (mg/L)	121.6 ± 74.4
White blood cell count (10^9^/L)	11.0 ± 3.3
Platelet count (10^9^/L)	179.3 ± 76.4
Hemoglobin (g/dl)	105.5 ± 12.7

Data are reported as number of patients (%) or mean ± SD. *Abbreviations*: *BMI* Body mass index, *COPD* Chronic obstructive pulmonary disease

### Microbiologic findings from wound culture

Microbiologic findings positive from wound cultures in 31 patients (79.5%), of which 22 patients had polymicrobial infection (Table 2). *Staphylococcus aureus* was the most common species (10.3%) of gram positive bacteria, while *Klebsiella pneumoniae* was the most common gram negative organism (41.0%), followed by Acinetobacter *baumanii* (33.3%) and *Escherichia coli* (25.6%). Fungal infections occurred in 4 cases.

**Table 2:** Microbiologic findings from wound culture in 39 patients with NSTIs

Isolated micro-organisms	n (%)
Wound culture, negative	7 (17.9)
Wound culture, positive	32 (82.1)
Monomicrobial infection	10 (25.6)
Polymicrobial infection	22 (56.4)
Aerobes (gram positive)	
*Staphylococcus aureus*	4 (10.3)
Enterococcus faecium	3 (7.7)
Enterococcus faecalis	2 (5.1)
Aerobes (gram negative)	
*Klebsiella pneumoniae*	16 (41.0)
Acinetobacter baumanii	13 (33.3)
*Escherichia coli*	10 (25.6)
Proteus mirabilis	6 (15.4)
Pseudomonas aeruginosa	5 (12.8)
Enterobacter cloacae	2 (5.1)
Fungi	
Candida albicans	3 (7.7)
Candida tropicalis	1 (2.6)

### Management and outcome of patients

Patient’s management and outcome were summarized in Table 3. All patients had a primary disease or injury in the abdominopelvic region. All patients underwent at least one debridement, abdominal washout and a fistula drain placement. Eight patients were treated with an open abdomen; negative pressure wound therapy was used in 33 patients and only 2 patients received hyperbaric oxygen therapy. In the 48 h following hospital admission, source control was achieved in 43.6% of patients. As the method of closure wound, 11 patients have undergone local skin grafting and 17 patients experienced direct suture. Forty one percent of these patients had delayed abdominal wall reconstruction. The intensive care unit (ICU) length of stay (LOS) was 19.7 ± 17.2 days, and an in-hospital LOS of 6.5 ± 5.8 days. The total in-hospital mortality was 38.5%.

**Table 3:** Management and outcome of patients

Variable	
Washing and drainage, n (%)	39 (100)
Multiple debridement (> 1), n (%)	17 (43.6)
Amputation, n (%)	2 (5.1)
Procedure, n (%)	
Open abdomen	8 (20.5)
NPWT	33 (84.6)
Hyperbaric oxygen	2 (5.1)
Source control< 48 h, n (%)	22 (56.4)
Wound closure, n (%)	
Local skin grafting	11 (28.2)
Direct suture	17 (43.6)
Delayed abdominal reconstruction, n (%)	16 (41.0)
In-hospital stay, mean (SD)	43.7 ± 44.9
ICU stay, mean (SD)	19.7 ± 17.2
In-hospital mortality, n (%)	15 (38.5)

Data are reported as number of patients (%) or mean ± SD. *Abbreviations*: *NPWT* Negative pressure wound therapy, *ICU* Intensive care unit

### Subgroup analysis

Participants in the traumatic group seem didn’t show worse basic characteristics compared with those in the non-traumatic group (Table 4). There was no statistical difference between traumatic and non-traumatic with regard to gender, BMI, severity of disease, treatment modality, wound culture, and hospital stay duration. All of the laboratory data on admission (CRP, WBC, platelet count, and hemoglobin concentration) were not significantly different between trauma and non-trauma patients. Compared to the non-trauma patients, the trauma individuals had younger age (*p* = 0.003), and less delayed abdominal reconstruction repair (*p* = 0.011), which suggest that differences in mortality rates in trauma setting were primarily confined to disease itself. There was a trend toward the prolonged ICU stay in patients who had non-trauma (*p* = 0.093). Of note, compared to trauma, non-trauma as a cause of GI fistula-associated NSTIs was associated with higher in-hospital mortality (*p* < 0.001).

**Table 4:** Characteristics of trauma and non-trauma patients

Variable	Trauma (*n* = 25)	Non-trauma (*n* = 14)	*P* value
Age (years), mean (SD)	41.2 ± 13.1	57.1 ± 18.5	0.003
Gender, (Male), n (%)	21 (84.0)	9 (64.3)	0.161
BMI (kg/m ^2^), mean (SD)	20.5 ± 2.0	20.3 ± 1.8	0.657
APACHE score on admission, mean (SD)	10.6 ± 3.0	12.9 ± 4.4	0.057
Shock on admission, n (%)	14 (56.0)	7 (50.0)	0.718
Positive blood cultures, n (%)	12 (48.0)	5 (35.7)	0.458
Multiple fistulas, n (%)	13 (52.0)	3 (21.4)	0.063
The Laboratory data on admission, mean (SD)			
C-Reactive Protein (CRP) (mg/L)	113.5 ± 76.7	136.0 ± 70.5	0.371
White blood cell count (10^9^/L)	11.2 ± 3.7	10.6 ± 2.4	0.615
Platelet count (10^9^/L)	191.7 ± 77.5	157.1 ± 71.9	0.178
Hemoglobin (g/dl)	106.1 ± 12.6	104.4 ± 13.2	0.683
Wound culture, n (%)			
Monomicrobial infection	7 (28.0)	3 (76.9)	0.652
Polymicrobial infection	15 (76.9)	7 (76.9)	0.546
Klebsiella pneumoniae	11 (44.0)	5 (35.7)	0.614
Fungi	2 (8.0)	2 (13.3)	0.535
Multidrug-resistant organisms	13 (52.00)	5 (35.7)	0.328
Management, n (%)			
Open abdomen	6 (24.0)	2 (14.3)	0.471
NPWT	23 (92.0)	10 (71.4)	0.088
Hyperbaric oxygen	2 (8.0)	0 (0)	0.277
Source control< 48 h, n (%)	16 (64.0)	6 (42.9)	0.201
Wound closure, n (%)			
Local flap	9 (36.0)	2 (14.3)	0.148
Direct suture	12 (48.0)	5 (35.7)	0.458
Delayed reconstruction repair, n (%)	14 (56.0)	2 (14.3)	0.011
In-hospital stay, mean (SD)	36.4 ± 27.4	56.9 ± 64.9	0.173
ICU stay, mean (SD)	16.3 ± 11.3	25.9 ± 23.6	0.093
In-hospital mortality, n (%)	7 (28.0)	7 (50.0)	< 0.001

Data are reported as number of patients (%) or mean ± SD. *Abbreviations*: *BMI* Body mass index, *APACHE* Acute physiology and chronic health evaluation score, *NPWT* Negative pressure wound therapy

Several variables differed from ICU survivors and nonsurvivors (Table 5). Male gender, older age and a higher APACHE II scores at admission were all associated with higher mortality (*p* = 0.028; *p* = 0.025; *p* = 0.013, respectively). Patients who died was more likely to multiple fistula (*p* = 0.010), and to suffer shock on admission than survivors (*p* = 0.054). The laboratory data on admission in survivors and non-survivors are presented in Table 5.The mean CRP on admission were higher in non-survivors than survivors on admission (*p* = 0.001). Similarly, the platelet count and hemoglobin concentration was lower in non-survivors on admission (*p* < 0.001; *p* = 0.001, respectively). The white blood cell count on admission was decreased in survivors and increased in those non-survivors, although the difference did not reach statistical significance (*p* = 0.126). Compare to survivors, non-survivors have more likely to found polymicrobial infection, *Klebsiella pneumoniae*, *fungi* and multidrug-resistant organisms from the wound culture (*P* = 0.003; *P* < 0.001; *P* = 0.008; *P* = 0.007, respectively).We also found no significant differences in in-hospital surgical management between survivors and non-survivors. However, source control< 48 h has significant differed between survivors and non-survivors (*p* = 0.022).

**Table 5:** Clinical data and outcome according to survival status in hospital

	Survivors (*n* = 24)	No-Survivors (*n* = 15)	*P*
Gender, (Male), n (%)	22 (91.7)	8 (53.3)	0.028
Age (years), mean (SD)	42.5 ± 13.4	54.9 ± 19.9	0.025
BMI (kg/m ^2^), mean (SD)	20.7 ± 2.1	20.0 ± 1.5	0.230
APACHE score on admission, mean (SD)	10.4 ± 3.0	13.5 ± 4.0	0.013
Etiology, n (%)			
Trauma	15 (62.5)	10 (66.7)	0.792
Tumor	5 (20.8)	4 (26.7)	0.674
Other	3 (12.5)	2 (13.3)	0.940
Shock on admission, n (%)	10 (41.7)	11 (73.3)	0.054
Positive blood cultures, n (%)	8 (33.3)	9 (60.0)	0.102
Multiple fistulas, n (%)	6 (25.0)	10 (66.7)	0.010
The Laboratory data on admission, mean (SD)			
C-Reactive Protein (CRP) (mg/L)	92.5 ± 70.3	173.4 ± 50.5	0.001
White blood cell count (10^9^/L)	12.0 ± 4.0	10.4 ± 2.7	0.126
Platelet count (10^9^/L)	217.6 ± 56.3	110.8 ± 57.9	< 0.001
Hemoglobin (g/dl)	96.9 ± 13.2	110.3 ± 9.7	0.001
Wound culture, n (%)			
Monomicrobial infection	8 (33.3)	2 (13.3)	0.164
Polymicrobial infection	9 (37.5)	13 (86.7)	0.003
Klebsiella pneumoniae	2 (8.3)	12 (80.0)	< 0.001
Fungi	0 (0)	4 (26.7)	0.008
Multidrug-resistant organisms	7 (28.0)	11 (73.3)	0.007
Management, n (%)			
Open abdomen	5 (20.8)	3 (20.0)	0.950
NPWT	21 (87.5)	12 (80.0)	0.528
Hyperbaric oxygen	1 (4.2)	1 (6.7)	0.731
Source control< 48 h, n (%)	17 (63.0)	5 (33.3)	0.022

Data are reported as number of patients (%) or mean ± SD. *Abbreviations*: *BMI* Body mass index, *APACHE* Acute physiology and chronic health evaluation score, *NPWT* Negative pressure wound therapy

### Cause of death

Fifteen of the 39 patients died in hospital. Evaluation of factors associated with in-hospital death is shown in Table 6. Multiple organ dysfunction syndrome, shock, polymicrobial infection, abdominal hemorrhage, multidrug-resistant organisms and source control failure were the main cause of in-hospital mortality. Abdominal hemorrhage occurred in nearly all the trauma patients who died (7/9); this may explain the overall mortality in the subgroup of trauma patients was not different from the mortality rate in the non-trauma population.

**Table 6:** Cause of death (*n* = 15)

Variable	n (%)
MODS	13 (86.7)
Source control failure	13 (86.7)
Shock	11 (73.3)
Polymicrobial infection	10 (66.7)
Abdominal hemorrhage	9 (60.0)
Multidrug-resistant organisms	9 (60.0)
Cardiac dysfunction	2 (13.3)

*Abbreviations*: *MODS* Multiple organ dysfunction syndromes

## Discussion

NSTIs are a group of aggressive soft tissue infections characterized by widespread necrosis of the fascia and subcutaneous tissue with initial sparing of the skin and muscle [24]. Options of the treatment of NSTIs depend on the etiology of the infection and anatomic location of the process. When NSTIs occur are in association with gastrointestinal fistulas, they are often associated with peritoneal contamination, intra-abdominal abscesses, fluid loss, and evisceration [11, 12, 25]. As previously noted, early identification of sepsis, source control, and prompt antibiotic administration remains the mainstay of treatment in abdominal wall necrotizing fasciitis [26].

**Fig. 2:**
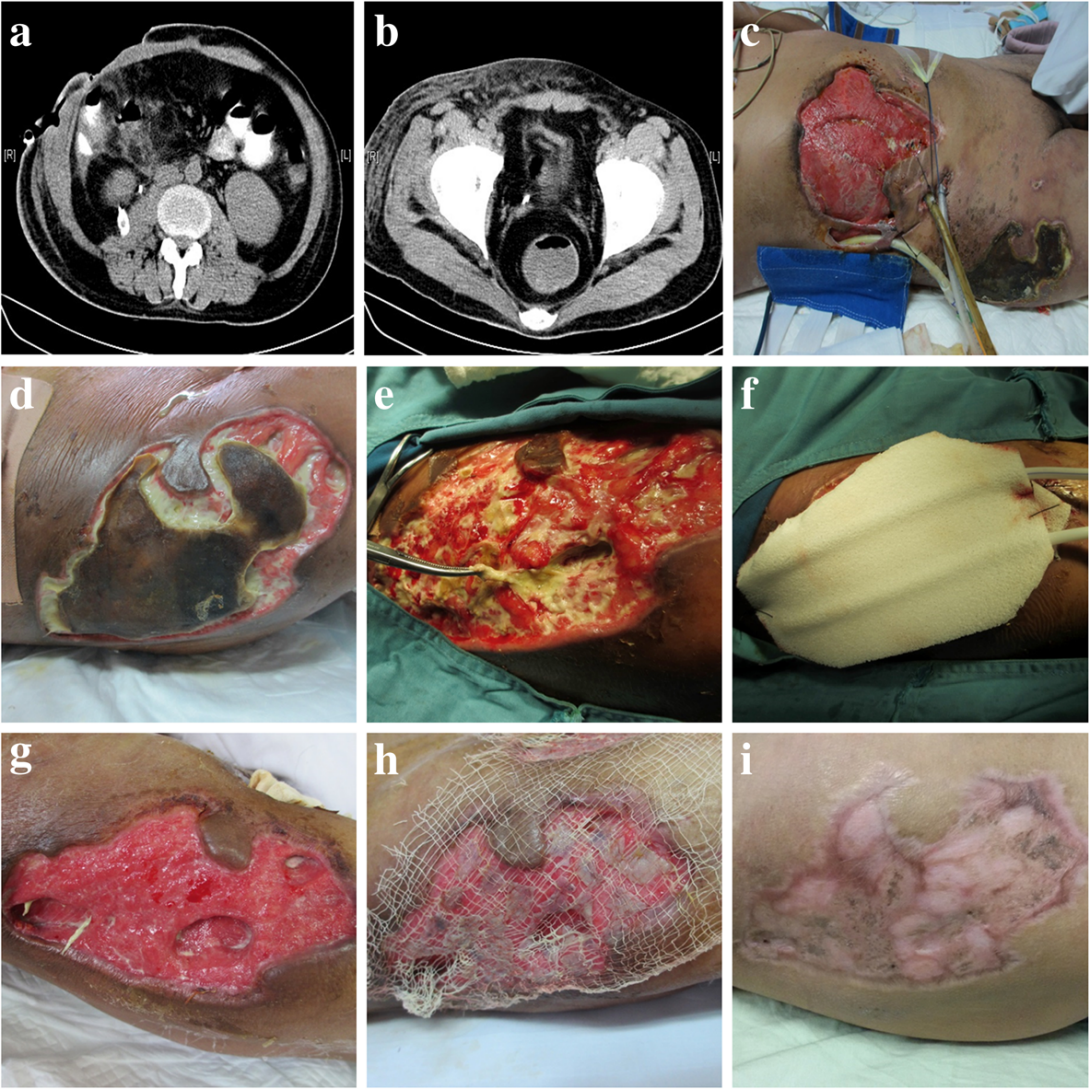
Case presentation. As shown by this figure, a 27-year-old male was taken up for traffic accident for polytrauma and the patient was successfully treated with fluid replacement, broad-spectrum antibiotics, and debridement of necrotic tissue, followed by reconstructive surgery. Computed tomography (CT) scans showed necrotizing soft tissue infection of the abdomen and perineum (**a**). Necrotizing had also affected the soft tissues below the skin, causing stretching of abdominal wall down to the right hip area (**b**). On local examination, the skin around the abdominal incision site was edematous, indurate and large area of skin defect in situ (**c** and **d**). Under local anesthesia, serial surgical debridement and change of regular dressing were performed at last 3 times a week. When the patient was taken for surgical debridement (**e**), cover the wound defect with chitosan sponge dressing to remove exudates and to promote wound healing (**f**), the severe infection subsided with daily wound irrigation and fresh granulation tissue gradually formed (**g**). At 32th day, her condition was significantly improved (**h**) and scar tissue eventually formed after discharge (**i**)

As shown by Fig. 2, a 27-year-old male was taken up for traffic accident for polytrauma and the patient was successfully treated with fluid replacement, broad-spectrum antibiotics, and debridement of necrotic tissue, followed by reconstructive surgery. Computed tomography (CT) scans showed necrotizing soft tissue infection of the abdomen and perineum (Fig. 2a). Necrotizing had also affected the soft tissues below the skin, causing stretching of abdominal wall down to the right hip area (Fig. 2b). On local examination, the skin around the abdominal incision site was edematous, indurate and large area of skin defect (Fig. 2c and d). Under local anesthesia, serial surgical debridement and change of regular dressing were performed at last 3 times a week. When the patient was taken for surgical debridement (Fig. 2e), cover the wound defect with chitosan sponge dressing to remove exudates and to promote wound healing (Fig. 2f), the severe infection subsided with daily wound irrigation and fresh granulation tissue gradually formed (Fig. 2g). At 32th day, her condition was significantly improved (Fig. 2h) and scar tissue eventually formed after discharge (Fig. 2i).

Different types of microorganisms can cause NSTIs. In our study, the majority of cases begin with an existing infection, most frequently in the abdomen, perineum or skin. Previous studies show mortality in patients with NSTIs was significantly associated with the presence of *Vibrio spp* in wound cultures and Streptococcus group A in blood cultures [27]. NSTIs caused by gastrointestinal fistula have special clinical characters compared with other origins. NSTIs in GI fistula patients have its own microbial distribution that all infections originated from the patients’ endogenous microflora. In the present study, *Klebsiella pneumoniae* were the most common pathogens isolated from wound culture, which is consistent with the results of previous studies [28]. Therefore, such high-risk groups should be given timely identification and bacteriologic diagnosis. Such patients will benefit from appropriate antibiotic administration. Further study is needed to determine the best combination strategy and optimum duration of antimicrobial therapy in these patients.

The key tasks in the surgical management of patients with NSTIs secondary to gastrointestinal fistula are source control. It is apparent that removal of infected necrotic tissue by drainage and debridement plays an important role in eliminating the trigger of an ongoing inflammatory response. It is borne in mind that intra-abdominal and extra-abdominal infections should receive the same attention. If the intra-abdominal disorders can be appropriately managed, the soft tissue infection also can be treated effectively [13]. However, some patients often have life-threatening conditions, such as hemodynamic instability and shock. When the clinician may first focus only on resuscitation, may result in a significant delay in diagnosis or an increase in clinical infection. Currently, open abdomen management became an effective treatment option of abdominal disaster, including intra-abdominal infections, gastrointestinal fistula, abdominal compartment syndrome (ACS) and wound dehiscence [29, 30]. Seternes et al. has reported 7 patients with necrotizing fasciitis undergo open abdomen treatment [31]. In our study, eight patients have undergone open abdomen to treat abdominal wall necrotizing fasciitis. Therefore, necrotizing fasciitis is an indication for open abdomen in some cases, such as difficult wound close and extensive tissue defects.

The use of NPWT in the wound management of NSTIs has been well documented. This technology facilitates the inflammation elimination and wound healing process, which involved mechanisms included reducing edema, removing infectious materials and exudates, and increasing blood supply [29, 32]. Our results are consistent with the positive outcomes of the previous studies. However, this method has some disadvantages and limitations such as abdominal skin loss after drainage and irrigation [33]. Additionally, skin margin necrosis due to excess tension in suture traction might be a potential complication. In our study, 11 patients had local skin grafting procedure and 16 patients underwent delayed abdominal wall reconstruction closure, because the defect size was reduced to small and medium after NPWT.

The mortality rate of NSTI is still high, and the overall mortality rate is between 25 and 73%, despite the use of modern powerful antimicrobial drug and advances in the nursing care [32, 34, 35]. Arif and her colleagues’ results showed that NSTIs-related deaths are associated with more diabetes and obesity when compared to other fatal diseases [32]. The other main prognostic factors of these patients include advanced age, poor nutrition, concomitant diseases and immunosuppressed host and nosocomial infection. However, it is quite different from surgical patients, especially in patients with GI fistula. After the appearance of fistula, continuous chemical irritation of digestive effluent can severely compromise skin integrity and exacerbate the spread of infection. In-hospital mortality in our cohort of patients was associated with multiple organ dysfunction syndrome, shock, polymicrobial infection, abdominal hemorrhage, multidrug-resistant organisms and source control failure. The above reasons are similar to the causes of death in patients with intra-abdominal infections [36], which is determined by the characteristics of our patients. Thus, any measure to prevent NSTIs development or control its drainage should be attempted. In our practice we treat all patients with washing and drainage, multiple debridement in 17 patients, open abdomen in 8 patients, NPWT in 33 patients and hyperbaric oxygen in patients. Finally, early source control (< 48 h) was achieved in 22 (56.4%) patients. This comprehensive strategy is also necessary to treat NSTIs.

Our research also has many disadvantages. As a retrospective study in single center, our patients are relatively complex surgical patients who have undergone multiple operative procedures and hospitalizations before NSTIs occurs, so that some of our pre-hospital information is incomplete. Furthermore, there are multiple infection sites of fistula combined with NSTIs, and the therapeutic effect of the drug may be confused. For example, the time of use of antibiotics and the effect of dosage on the final outcome of patients. In addition, we only focus on patients with surgical infections, which may make it difficult for our research to be extended to all clinical scenarios. However, at present, there are limited researches on NSTIs secondary to GI fistulas, and our study might have some implications for this special populations.

## Conclusion

The management of GI fistulas associated NSTIs challenge due to the presence of primary disease and the formation of fistulas, as well as variations in host system responses. Sepsis is the most important factor associated with the death of GI fistulas combined with NSTIs. Therefore, it is crucial to prevent the occurrence and deterioration of sepsis from the early source control. Our data may assist providing enlightenment for quality improvement in these special populations.

## Data Availability

The datasets used and analyzed in the current study are available from the corresponding author on reasonable request.

## Abbreviations

CRP: C-reaction protein
CT: Computed tomography
GI fistula: Gastrointestinal fistula
ICU: Intensive care unit
LOS: Length of stay
NPWT: Negative pressure wound therapy
NSTIs: Necrotizing soft tissue infections
SD: Standard deviation
WBC: White blood cells
